# The Unique Molecular and Cellular Microenvironment of Ovarian Cancer

**DOI:** 10.3389/fonc.2017.00024

**Published:** 2017-02-22

**Authors:** Thomas Worzfeld, Elke Pogge von Strandmann, Magdalena Huber, Till Adhikary, Uwe Wagner, Silke Reinartz, Rolf Müller

**Affiliations:** ^1^Institute of Pharmacology, Biochemical-Pharmacological Center (BPC), Philipps University, Marburg, Germany; ^2^Department of Pharmacology, Max-Planck-Institute for Heart and Lung Research, Bad Nauheim, Germany; ^3^Experimental Tumor Research, Clinic for Hematology, Oncology and Immunology, Center for Tumor Biology and Immunology, Philipps University, Marburg, Germany; ^4^Institute of Medical Microbiology and Hygiene, Biomedical Research Center, Philipps University, Marburg, Germany; ^5^Institute of Molecular Biology and Tumor Research, Center for Tumor Biology and Immunology, Philipps University, Marburg, Germany; ^6^Clinic for Gynecology, Gynecological Oncology and Gynecological Endocrinology, University Hospital of Giessen and Marburg (UKGM), Marburg, Germany; ^7^Clinic for Gynecology, Gynecological Oncology and Gynecological Endocrinology, Center for Tumor Biology and Immunology (ZTI), Philipps University, Marburg, Germany

**Keywords:** tumor microenvironment, ascites, tumor-associated macrophage, T cell checkpoints, extracellular vesicles, invasion, STAT, NFκB, adhesion, metastasis, mesothelial cell, T cell

## Abstract

The reciprocal interplay of cancer cells and host cells is an indispensable prerequisite for tumor growth and progression. Cells of both the innate and adaptive immune system, in particular tumor-associated macrophages (TAMs) and T cells, as well as cancer-associated fibroblasts enter into a malicious liaison with tumor cells to create a tumor-promoting and immunosuppressive tumor microenvironment (TME). Ovarian cancer, the most lethal of all gynecological malignancies, is characterized by a unique TME that enables specific and efficient metastatic routes, impairs immune surveillance, and mediates therapy resistance. A characteristic feature of the ovarian cancer TME is the role of resident host cells, in particular activated mesothelial cells, which line the peritoneal cavity in huge numbers, as well as adipocytes of the omentum, the preferred site of metastatic lesions. Another crucial factor is the peritoneal fluid, which enables the transcoelomic spread of tumor cells to other pelvic and peritoneal organs, and occurs at more advanced stages as a malignancy-associated effusion. This ascites is rich in tumor-promoting soluble factors, extracellular vesicles and detached cancer cells as well as large numbers of T cells, TAMs, and other host cells, which cooperate with resident host cells to support tumor progression and immune evasion. In this review, we summarize and discuss our current knowledge of the cellular and molecular interactions that govern this interplay with a focus on signaling networks formed by cytokines, lipids, and extracellular vesicles; the pathophysiologial roles of TAMs and T cells; the mechanism of transcoelomic metastasis; and the cell type selective processing of signals from the TME.

## Biological and Clinical Features of Ovarian Carcinoma

Ovarian cancer is the deadliest of all gynecological malignancies with >60,000 new cases reported annually in the United States and the European Union (1). More than 90% of malignant ovarian tumors are carcinomas, presumably originating from the ovarian surface epithelium and/or the fallopian tube. Ovarian cancer has a dire prognosis with an overall 5-year survival rate of <40%. The WHO classification distinguishes six major entities of ovarian tumors, i.e., serous, mucinous, endometrioid, clear cell, transitional cell, and squamous carcinoma (1), with high-grade serous ovarian carcinoma (HGSOC) representing the most common subtype. The majority of patients present with advanced stage disease and tumor masses in the abdomen beyond the pelvis, contributing to the disastrous prognosis of the disease. HGSOC is characterized by a very high frequency of *TP53* mutations (97%), germline and somatic *BRCA1/2* mutations (~40%), as well as amplification and overexpression of *MYC* (>50%) (2).

According to the prevailing opinion, HGSOCs arise from the fimbriated fallopian tube epithelium (3). There is some evidence to suggest that serous tubal intraepithelial carcinomas (STICs) are precursor lesion of HGSOC, although recent evidence obtained by next-generation sequencing suggests that lesions histologically identified as STICs may actually represent micrometastases (4). Several features contribute to the fatal nature of HGSOC, which distinguish it from other human cancers, in particular, the role of the peritoneal fluid in cancer cell spread:

- Tumor cells can be shed at a very early stage of the disease. Even at a stage when the primary tumor is still confined to the ovary, cancer cells can be detected in peritoneal lavage fluid.
- Besides hematogenous dissemination to the omentum (5), the spread of tumor cells to other pelvic and peritoneal organs is facilitated by the peritoneal fluid serving as a carrier (6). This transcoelomic dissemination is a major route for the adhesion of cancer cells to the omentum and serous membranes lining the peritoneal organs, giving rise to metastatic lesions growing into the peritoneal cavity rather than invading through the lamina propria (6, 7).
- The peritoneal environment, which is frequently formed by the effusion building up in the peritoneal cavity (ascites), is rich in tumor-promoting soluble factors (8), extracellular vesicles (9), highly tumorigenic cancer cells (10), and different types of immune cells, including large numbers of different types of T cells (11), tumor-associated macrophages (TAMs) (12, 13), and other host cells, supporting tumor cell proliferation, progression, chemoresistance, and immune evasion (14–16).
- In contrast to most other cancers, metastases at distant sites are confined to late stages (6). The most serious problem for most HGSOC patients is recurrent, aggressive growth of metastatic lesions within the peritoneal cavity.

### Mechanisms of Therapy Failure

Although HGSOC is typically highly sensitive to chemotherapy, a small subgroup (<10%) is refractory to first-line therapy, pointing to a mechanism of inherent resistance. However, even after a clinical remission, most patients suffer from a relapse of the disease (1). While some of these patients are refractory to chemotherapy due to acquired chemoresistance, the majority undergo remission under the same treatment regimen. This regrowth of lesions displaying a similar chemosensitivity as the primary disease points to a mechanism of therapy failure that is fundamentally different form intrinsic or acquired resistance. However, the mechanisms underlying this transient chemoresistance are unknown.

A number of studies have associated chemoresistance with epithelial–mesenchymal transition (EMT), cell cycle arrest, blocked apoptosis, drug efflux, and several signaling pathways, including TGFβ, WNT, and NOTCH, but these observations did not yield a deep understanding of the mechanisms leading to relapse of the disease (17). It has also been a topic of intense research to clarify whether the regrowth of tumors after a complete clinical response is caused by a small population of cancer stem cells that are endowed with stem-like properties (18–20). However, multiple studies showed that ovarian cancer cell subpopulations express stemness markers at highly variable levels in different combinations and with none of these markers being obligatory (21–26). These findings suggest that a common or early ovarian cancer stem cell may not exist or has not been identified yet.

Comprehensive genomic studies by The Cancer Genome Atlas (TCGA) consortium have confirmed the prevalence of the genetic alterations described earlier and identified a number of recurrent, but infrequent changes (2). A more recent study has identified PTEN loss as another common driver event associated with a poor prognosis (27). This study also defined four transcriptional subtypes of ovarian carcinoma (differentiated, proliferative, immunoreactive, and mesenchymal), which were, however, not associated with differences in survival. In contrast, a recent reanalysis of the TCGA data indicated a favorable outcome for the immunoreactive subtype, presumably due to partial antitumor immune response (28). However, new insights into the precise mechanisms of chemoresistance were not gained by these studies. Whole-genome sequencing of DNA from patients with primary refractory, sensitive, and matched acquired resistant disease suggested that (i) inactivation of the tumor suppressor genes *RB1, NF1, RAD51B*, and *PTEN*, (ii) reversions of germline *BRCA1/2* mutations or loss of *BRCA1* promoter methylation, and (iii) overexpression of *MDR1* are associated with, and may contribute to, acquired chemotherapy resistance (29). In contrast, amplification of *CCNE1* was frequently observed in primary refractory cancer. However, these findings have not been linked to therapy failure or chemoresistance. Finally, a microarray study published in 2005 (30) identified an 11-gene signature characteristic of relapsed HGSOC, but mechanistic links to therapy resistance remain hypothetical.

It has been suggested that treatment failure is caused, in part, by cells escaping chemotherapy-induced cell death by entering a transient state of resistance, e.g., by entering a non-cycling state with low metabolic activity. This is a characteristic feature of detached tumor cells and spheroids floating in the peritoneal fluid or ascites. However, tumor cells surviving chemotherapy can trigger disease recurrence only if they are able to invade the peritoneum or omentum to establish proliferative lesions. Therefore, cancer cell adhesion to, and invasion of, the mesothelial cell layer lining these organs, and thus the mechanisms of transcoelomic dissemination appear to be central aspects of therapy failure. Conditional resistance can also be modulated by interactions with host cells (31–33), whose presence and functional states are spatially and temporally highly heterogeneous.

## The Cellular Microenvironment and Cytokine Signaling Network of Ovarian Cancer Ascites

Ascites-associated cancer cells occur as single cells or multicellular spheroids and are likely to be responsible for peritoneal dissemination and to contribute to relapse of the disease (34). Besides these tumor cells, macrophages and T cells are the most common cell types in ovarian carcinoma-associated ascites, but other host cells are also present to a variable extent, including natural killer (NK) cells, fibroblasts, adipocytes, and mesothelial cells (15, 35, 36). Cytokines and growth factors released by these cells into the tumor microenvironment (TME) play essential roles in tumor growth and progression, cancer dissemination, and immune escape (12, 15, 36–40). This is exemplified by the induction or promotion of

(i) cell proliferation and survival, for example, by EGF family members, interleukin (IL)-6, and TGFβ (41);
(ii) EMT, cancer cell invasion, and metastasis, in particular, through TGFβ (42);
(iii) stemness by KIT ligand and R-spondins as ligands for CD117 (21) and LGR5 (43, 44), respectively.
(iv) angiogenesis by vascular endothelial growth factor (VEGF), basic FGF, and CXCL8/IL-8;
(v) immune cell migration to the tumor by chemokines of the CCL and CXCL families (45);
(vi) immune cell suppression, for example, by VEGF, IL-6, IL-10, LIF, and TGFβ (46); and
(vii) the accumulation of ascites, mainly though the action of VEGF as a vascular permeability factor (47).

Several lines of evidence support the clinical significance of these mediators. Thus, evaluation of genomic data has identified a number of clinical associations of signaling loops established by polypeptide ligands and their receptors in solid tumor tissue of advanced ovarian cancer, including TGFβ, PDGF, VEGF, ephrin, CXCL12, and CCL chemokines (25). Furthermore, several studies have demonstrated highly significant associations between the ascites levels of various cytokines and the relapse-free survival (RFS) or overall survival (OS) of ovarian cancer patients, for example, TGFβ, IL-6, IL-10, and LIF (12, 40, 48–52).

**Figure 1:**
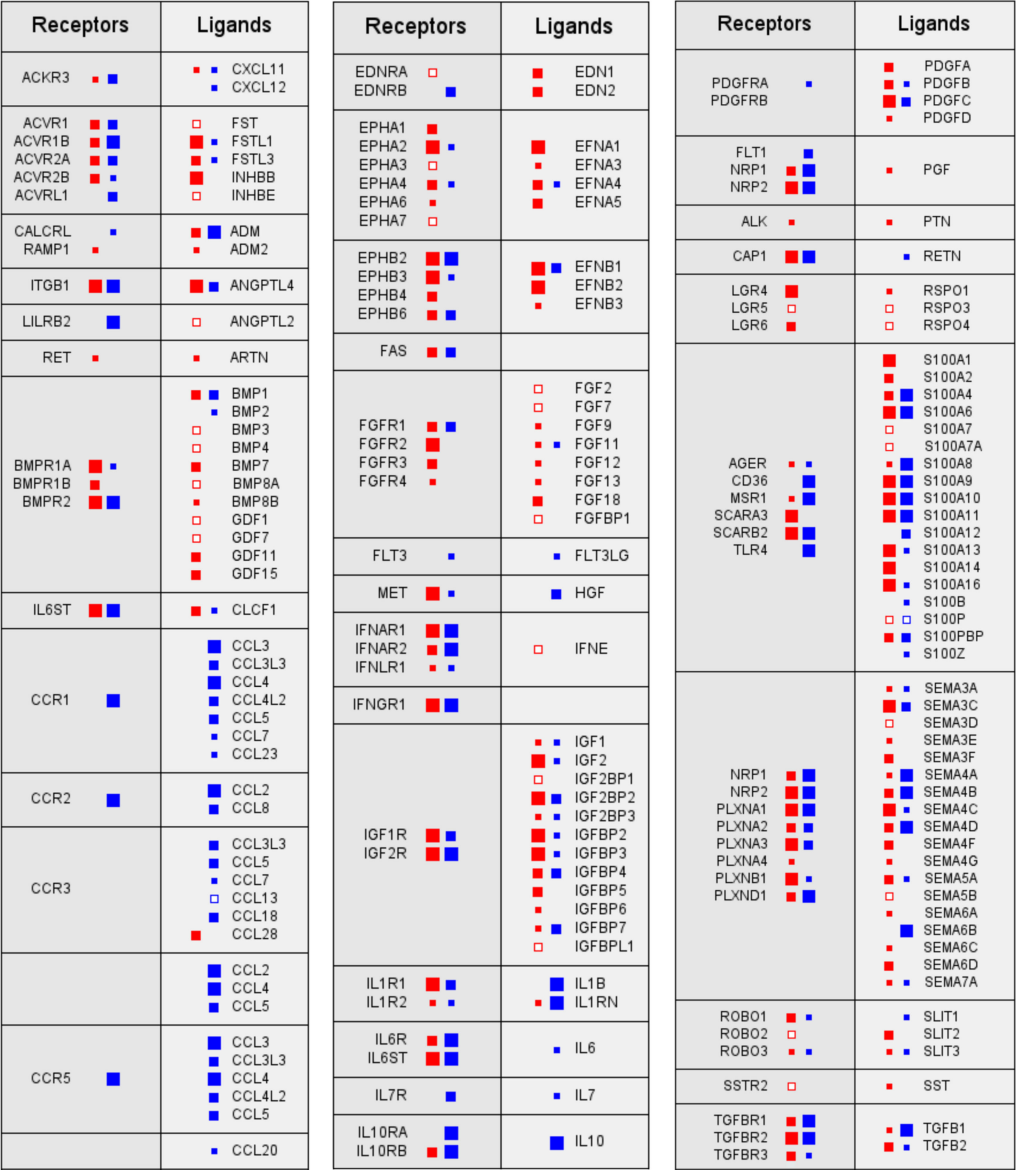

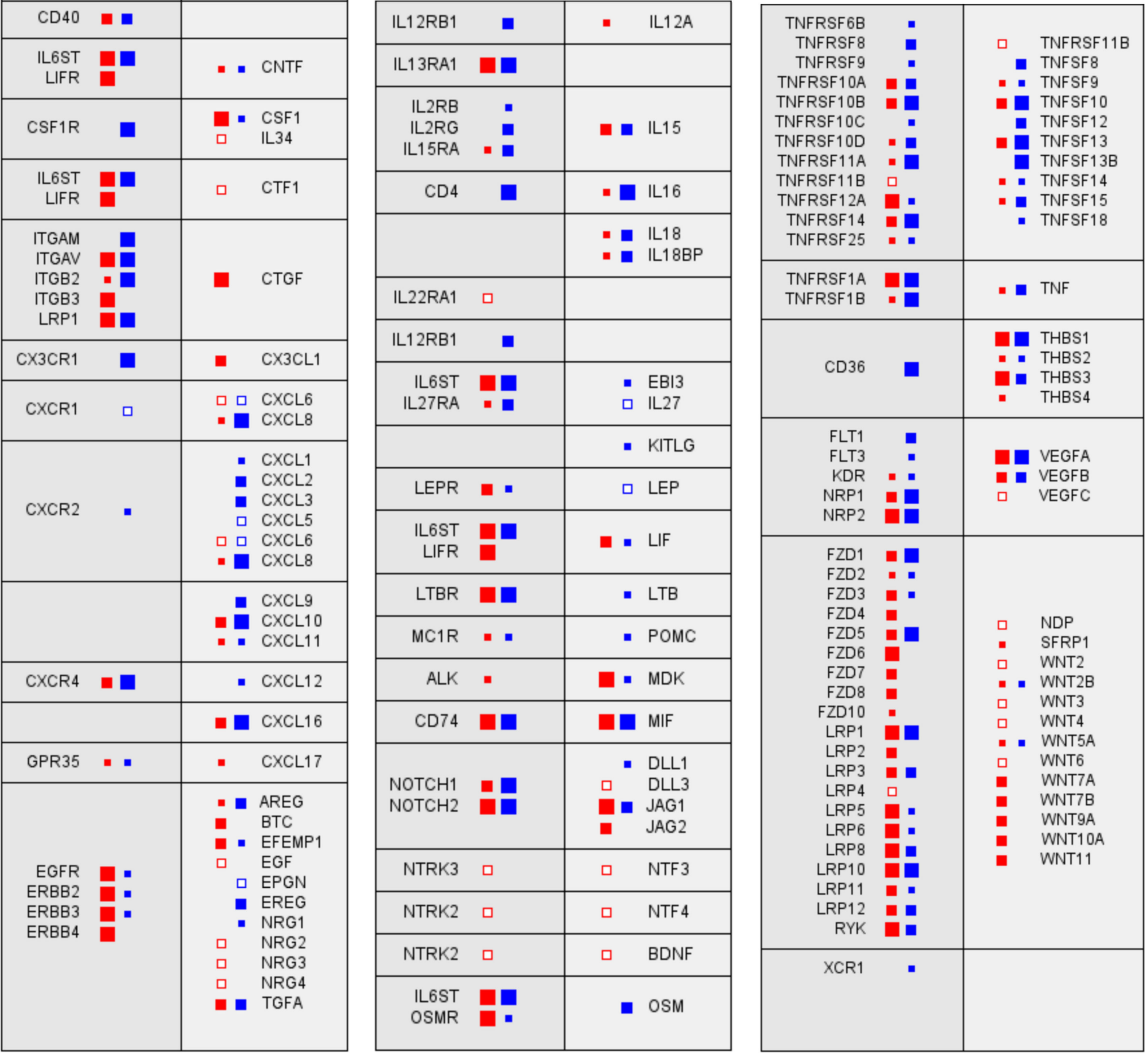
**Signaling network between tumor cells and tumor-associated macrophages (TAMs) in ovarian cancer ascites**. Prominent examples of signaling pathways operating between tumor cells (red) and TAMs (blue). Genes for receptors and their cognate ligands are ordered in adjacent blocks on the left and right sides, respectively, of each box. The sizes of the filled squares indicate the level of expression determined by RNA-Seq (large: median TPM > 50; intermediate: TPM > 10; and small: TPM > 0.3) (52). Open squares indicate cases, where substantial expression (TPM > 3) was observed only in a fraction of samples (>10%).

Despite their undisputed role within the TME, the reciprocal interactions of tumor and host cells *via* soluble mediators are only partially understood. A recent study has addressed this issue by determining the transcriptome for tumor cells and TAMs from ovarian cancer ascites samples and using these data to establish an extensive network of signaling mediators and their receptors and thereby the first global overview of the origins and targets of cytokines in the TME (Figure 1). This study defined multiple signaling pathways between tumor cells and TAMs as well as cell type restricted, autocrine mechanisms and uncovered new clinically relevant components within this signaling network (52).

The multifaceted cooperation of tumor cells and TAMs is exemplified by signal transducer and activator of transcription 3 (STAT3)-inducing cytokines IL-10, IL-6, and LIF, all of which are predictive of a poor survival: while the major source and target of IL-10 are TAMs, LIF is synthesized by, and acts on, tumor cells, whereas IL-6 production and action are not cell type selective (52). Both tumor cells and TAMs also contribute to an extensive TGFβ-signaling network, consistent with previous findings (42, 53, 54). Multiple components of these pathways are associated with a short RFS, including TGFβ1, TGFβ3, and TGFβR1, the former produced most strongly by TAMs (52). Furthermore, WNT signaling through frizzled receptors also plays a major role, with WNT7A and WNT11 deserving particular attention in light of strong associations with a poor survival. The same study also revealed a comprehensive network of axon guidance molecules, with SEMA5A, SEMA6C, and the ephrin receptor gene EPHB2 standing out as strong indicators of a poor RFS. On the other hand, the identification of all three components, the norrin–frizzled 4–TSPAN12 complex (55), as indicators of a longer RFS is particularly intriguing, since this finding points to an unexpected role for the frizzled ligand norrin in tumor suppression (52).

There is impressive progress in understanding the molecular networks regulating TAMs; however, the role of other host cells within this signaling network is even less understood, including myeloid-derived suppressor cells (MDSCs), different types of T cells, NK cells, adipocytes, and fibroblasts, which are clearly important constituents of the TME. It is also unknown how ascites-associated tumor and host cells differ from their counterparts in solid tumor masses. Therefore, the next obvious step is to extend this work to other cell types of ovarian cancer ascites and solid tumor tissue to obtain a complete integrated picture of the cytokine signaling network operating in the ovarian cancer TME.

## The Lipid Signaling Network of Ovarian Cancer Ascites

Molecules generated by the cleavage of phospholipids and present in malignant effusions represent another important class of soluble cancer-promoting mediators. The first major group of lipid mediators highly relevant in the context of ovarian cancer ascites is represented by polyunsaturated fatty acids (PUFAs), in particular eicosanoids (56–58), including arachidonic acid (AA) and its metabolites. These include prostanoids, hydroxyeicosatetraenoic acids, and leukotrienes that are derived from AA by enzymatic cascades initiated by either cyclooxygenases or lipoxygenases. Prostaglandin E_2_ (PGE_2_), in particular, is known to promote tumor progression as an immune suppressor and trigger of angiogenesis (59). The PGE_2_, PGI_2_, and LTB_4_ pathways have been linked with clinical progression through the PGE_2_ receptor PTGER3 on tumor cells, expression of the PGI_2_-synthesizing enzyme PTGIS by tumor cells and TAMs, as well as release of LTB_4_ into the TME by both cell types (52).

Non-metabolized AA itself is also present in ovarian cancer ascites. Its concentrations are inversely linked to RFS (52), which is presumably functionally linked to the AA-generating phospholipase PLA_2_G_7_ (see below). It is possible that these associations are not (only) due to AA-derived metabolites, since AA can act directly on intracellular proteins, including PKC and peroxisome proliferator-activated receptors (PPARs) (60–63), in particular at the high concentrations of up to 50 μM in ascites (52). Linoleic acid, another PUFA present at even higher levels in ovarian cancer ascites, did not show any survival associations, but was shown to deregulate target genes of PPARβ/δ by acting as an endogenous agonistic ligand (63). Intriguingly, several of these genes are linked to protumorigenic functions. These include *ANGPTL4*, which is linked to a poor clinical outcome of ovarian cancer (63).

The second major group of lipids found in ascites encompasses lysophosphatidic acids (LPA), a mixture of lipids with different ester- or ether-linked fatty acids in the sn1 position (64–69). Extracellular LPA is generated from phospholipids by the consecutive action of two enzymes, i.e., secretory phospholipases A_2_ and the lysophospholipase D autotaxin (67, 69). LPA production is counteracted by lipid phosphatases of the LPPR family (70), although LPA degradation seems to play an insignificant role in ovarian cancer ascites based on the low expression of *LPPR* genes in both tumor cells and TAMs. TAMs seem to play an essential role in phospholipid cleavage as a major source of PLA_2_G_7_ and autotaxin, the former being associated with a short RFS (52). On the other hand, it has been reported that mesothelial cells display strong PLA_2_ activity and may thus significantly contribute to LPA production (71). Correlative studies have demonstrated that high levels of LPA correlate with poor prognosis (64, 72–75). LPA promotes cancer cell survival and multiple steps of the metastatic cascade, including adhesion, migration, and invasion of ovarian cancer cells (76–87).

LPA interacts with six different G-protein-coupled membrane receptors (LPARs) (69) that are expressed by tumor cells and TAMs in a highly cell type-selective fashion (52). This is intriguing in view of the fact that different receptors respond to different LPA species and trigger distinct, partly overlapping signaling pathways, including cAMP-PKA, PI_3_K-AKT, Ras-MAPK, PLC-Ca^2+^-PKC, and RHO-ROCK (69) to promote cancer cell invasion, chemoresistance, and tumor progression (66–68, 71, 88, 89). The role of LPA on TAMs or other host cells is unknown. Different LPA species have been detected in ovarian cancer ascites (64, 90), but associations of distinct LPAs with specific biological features or clinical outcome have not been investigated.

## Extracellular Microvesicles (EVs) in Ovarian Cancer Ascites

The complex network of cellular interactions and soluble factors, which promotes tumor growth, metastasis, immune evasion, and drug resistance in ovarian cancer, is also supported by EVs. EVs are released by tumor cells and normal healthy cells and mediate the transfer of proteins, lipids, and nucleic acids between malignant and non-malignant cells to alter the function and phenotype of the recipient cell. EVs are either shed from the cell surface or released as exosomes *via* exocytosis upon generation in multivesicular bodies. Signal transmission *via* EVs adds another level of complexity to the cell communication network as EVs simultaneously release multiple molecules impinging on signaling pathways in the recipient cell. Molecules previously not considered to be exchanged between cells, including mRNA and microRNAs (miRNAs), are transferred by EVs.

microRNAs are non-coding RNAs that control target genes posttranscriptionally. The dysregulation of miRNAs is involved in the tumorigenesis of ovarian cancer. Thus, exosomal miRNAs isolated from the serum or ascites fluid are probably of clinical relevance and may serve as diagnostic markers, as reviewed by Nakamura et al. (91). The first study profiling the miRNA content of exosomes isolated from patient serum identified a disease-specific miRNA signature with exosomal miRNAs (miR-21, miR-141, miR-200a, miR-200b, miR-200c, miR-203, miR-205, and miR-214) (92). Several follow-up studies demonstrated that miRNAs not only reflect diagnostic and prognostic biomarkers in ovarian cancer but also serve as predictors for response to therapy and represent potential therapeutic targets. This topic has been covered in depth by a recently published review (93).

**Figure 2:**
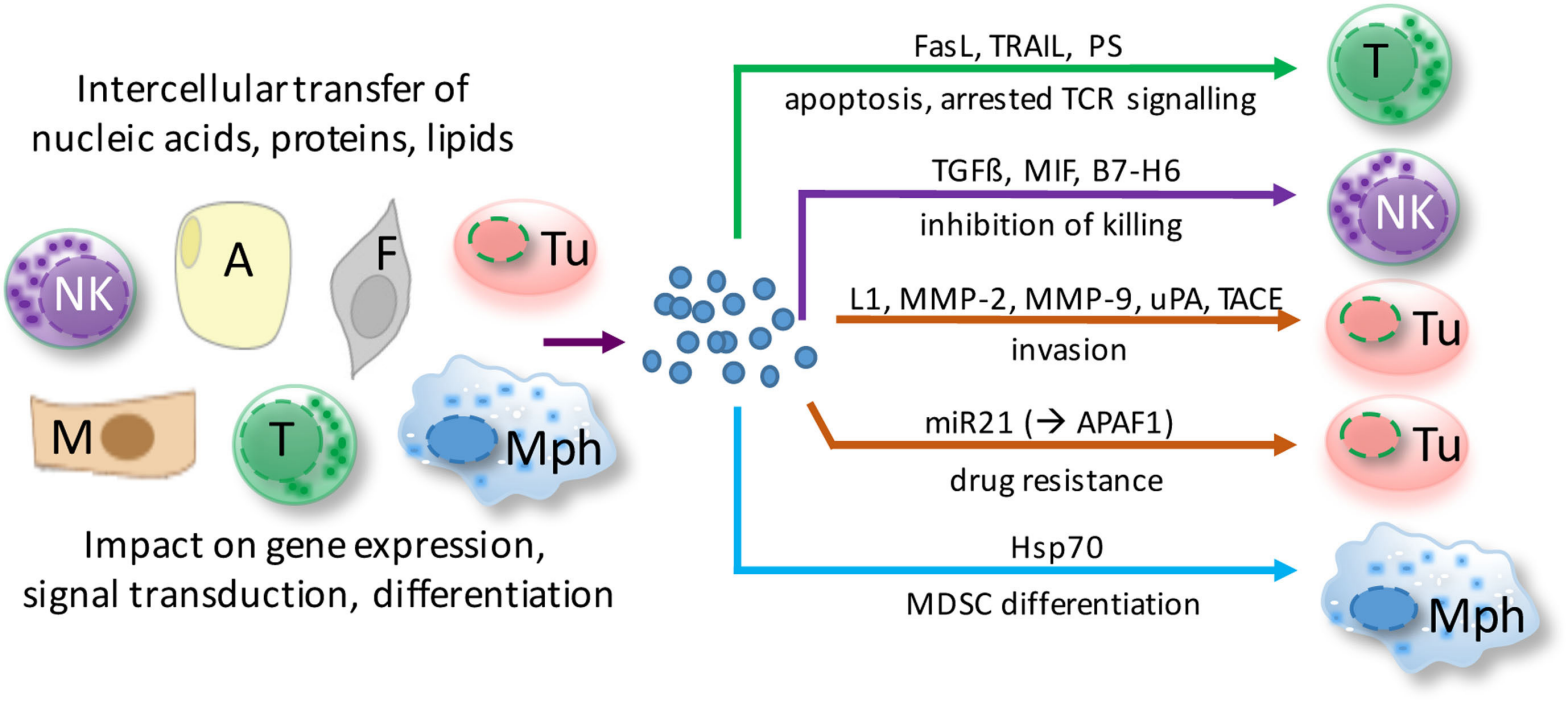
**Functions of extracellular microvesicles (EVs) in ovarian cancer ascites**. EVs are released by virtually all cell types of the tumor microenvironment and shape cellular functions of both tumor cells and host cells *via* different pathways. Depicted examples affect major hallmarks of ovarian cancer to promote tumor growth and metastasis. A, adipocyte; F, fibroblast; M, mesothelial cell; Mph, macrophage; NK, natural killer cell; T, T cell; Tu, tumor cell.

Even though our knowledge of the molecular composition, biological functions, and cellular sources of EVs in ovarian cancer is still limited, available evidence suggests that EVs in ascites interfere with immune evasion, invasion, and drug resistance (Figure 2). The following paragraphs highlight recent data suggesting a role for ascites EVs in ovarian cancer pathogenesis.

### Role of MVs in Immune Evasion

Several studies investigated the immunomodulating potential of EVs derived from different cell types. Strikingly, EVs released by dendritic cells (DCs) and B cells reveal antitumor and immune stimulatory effects and can provide costimulatory proteins that are able to activate T cells when presented with tumor antigens (94). Based on these observations, DC-EV-based antitumor vaccination studies were developed and tested in clinical trials (95–97).

On the contrary, exosomes secreted from ovarian tumor and bystander cells suppress the immune response (9). EVs collected from ovarian ascites impaired the cytotoxic activity of peripheral lymphocytes and induced apoptosis of lymphocytes and DCs. This correlated with the vesicle-associated expression of FAS ligand (FAS-L) and TRAIL, both known to be involved in apoptosis induction of immune cells. In line with these findings, secreted vesicle-associated FAS-L purified from patient ascites could trigger FAS-mediated apoptosis in a T cell line (98). These results suggest that vesicle-associated FAS-L counterattacks Fas-bearing immune cells including T cells, thus promoting immune evasion to support tumor cell survival. Moreover, there is evidence that ovarian ascites EVs can inhibit T cell activation by arresting the T cell-signaling cascade (99). This was mechanistically attributed to phosphatidylserine (PS), which is present on EVs. However, the functional role of PS remains to be confirmed since this molecule is required (but not sufficient) for the uptake of EVs by recipient cells (100).

Extracellular microvesicles do not only interact with cytotoxic lymphocytes but also regulate the activation of MDSCs *via* vesicle-associated HSP70, which engages toll-like receptor (TLR) 2 on MDSC (101). There is experimental evidence that these mechanisms might provide a target for successful immunotherapies. Depletion of HSP70-positive EVs and blockade of the HSP70/TLR2 interaction using a specific peptide aptamer diminished the activation and number of MDSCs significantly and inhibited tumor progression in tumor-bearing C57Bl/6 mice. Thus, targeting or interfering with EVs holds promise for novel immunotherapies to treat ovarian cancer. However, the development of innovative therapeutic concepts requires a more detailed understanding of EV biogenesis, molecular composition, and the mechanisms of target cell interaction.

### Role of EVs in Invasion

Most studies on ovarian cancer ascites EVs are related to their impact on immune evasion, but there is also evidence that EVs regulate invasion and metastasis. First, ovarian cancer EVs carry molecules that directly regulate tumor cell migration in an autocrine or paracrine fashion, including soluble L1 (102), CD24, EpCAM (103), and soluble ALCAM (104). Second, membrane vesicles from malignant ascites were also found to contain the activated proteases: matrix metalloproteinase (MMP)-2, MMP-9, uPA (105), and ADAM17/TACE (104). These vesicle-associated proteases promote extracellular matrix (ECM) degradation directly or through the release of soluble factors from the EVs, which may allow tumor cell invasion into the stroma to facilitate metastasis. Third, EVs may carry miRNAs, which interfere with invasion and migration either positively or negatively. One prominent example is the transfer of miR-6126, which targets integrin β1, a key regulator of cancer cell metastasis. The transfer of miR-6126 mimic to endothelial cells (ECs) reduced invasion and migration of ovarian cancer cells *in vitro* and tumor growth *in vivo* (106).

### EVs and Drug Resistance

The loading of EVs with specific miRNAs and the transfer of these miRNAs to recipient cells may also affect drug resistance. The vesicle-mediated transfer of miR21 from omental cancer-associated adipocytes and cancer-associated fibroblasts (CAFs) to ovarian cancer cells was recently identified as a pathway causing resistance to taxane-based chemotherapy. In the recipient cancer cells, miR-21 directly targets *APAF1* mRNA, which is indispensable for the initiation of apoptosis in response to taxanes (32, 107).

Taken together, the vesicle-mediated cross talk between cells of the ovarian cancer ascites emerges as a pivotal component that establishes a protumorigenic environment promoting—but probably not limited to—immune suppression, invasion, and drug resistance. It remains a challenge to gain a better understanding of the mechanisms regulating cargo sorting into vesicles and their biosynthesis to establish links between vesicle-associated signatures, effects on signaling pathways, and altered functions in recipient cells. This knowledge is a prerequisite to be able to develop new therapeutic options for ovarian cancer by interfering with vesicle biogenesis, loading with specific cargo or vesicle uptake.

## Impairment of Innate Immune Cells

The complex immune suppression network that effectively neutralizes antitumor immunity is regarded as one of the main reasons for disease progression and treatment failure. The activity of immune effector cells present within the TME, including CD4 T cells, CD8 T cells, and NK cells, is inhibited not only directly by tumor cells but also by immunesuppressive T regulatory cells (Tregs), immature DCs, MDSCs, and TAMs (15, 16, 37). The antitumor activity of these cells is mediated by the concerted action of a plethora of mediators comprising MVs, cytokines (such as IL-10 and TGF-β), lipids (for example, PGE_2_), secreted enzymes (e.g., indolamin-2,3-dioxygenase and arginase), and membrane-bound ligands [including B7-H1 and programmed cells death protein 1 (PD-1)].

### Tumor-Associated Macrophages

**Figure 3:**
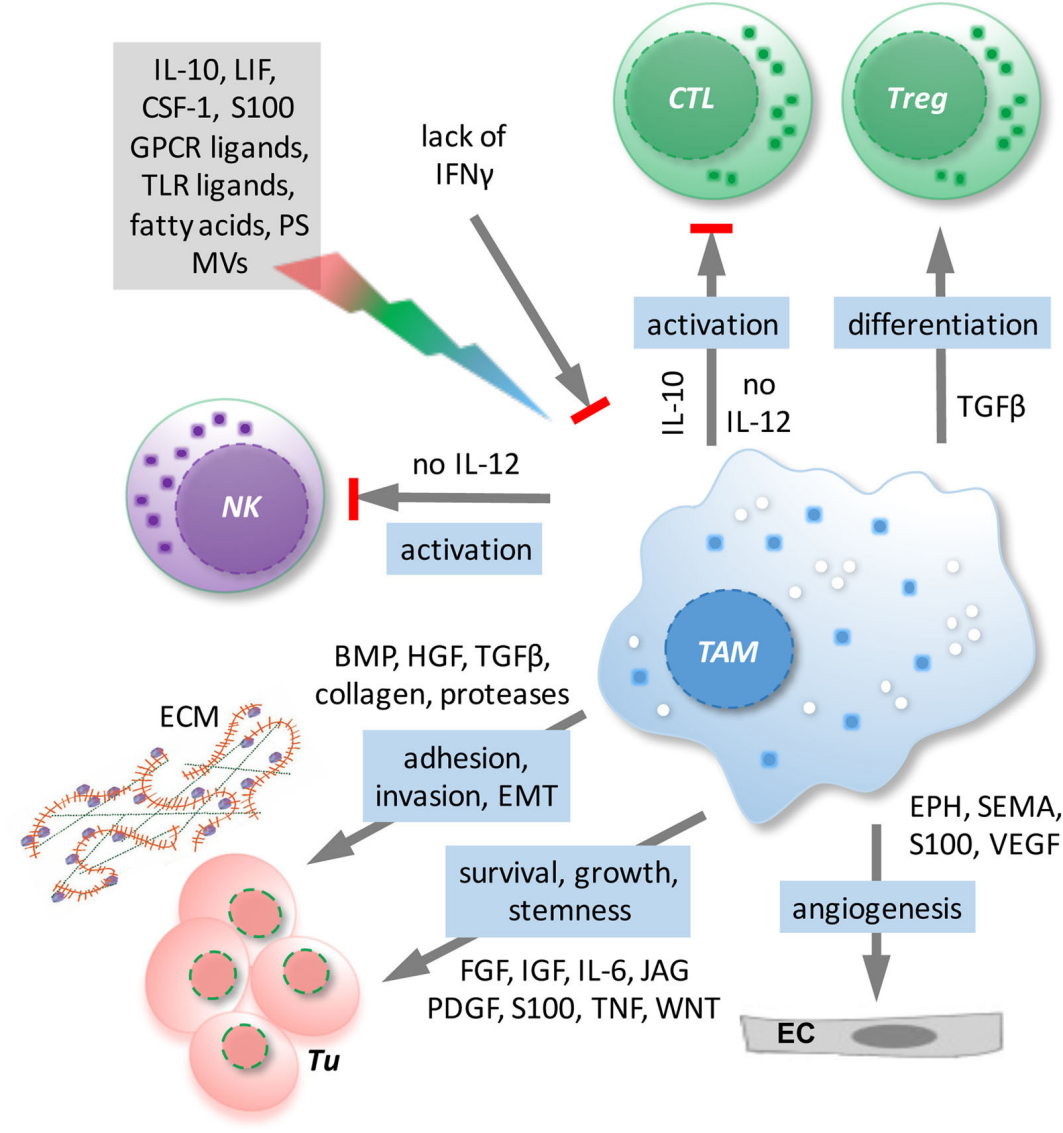
**Functions/dysfunctions of tumor-associated macrophages (TAMs) in the ovarian cancer tumor microenvironment (TME) and examples of major mediators of these functions**. Multiple mediators (gray box) in the TME as well as the lack of interferon (IFN)γ determine the activation state and function of TAMs. In response to these triggers, TAMs produce a plethora of soluble factors impinging on tumor cells and other host cells and block the expression of essential antitumor mediators such as interleukin (IL)-12.

Tumor-associated macrophages represent the major subpopulation of myeloid cells in ovarian cancer ascites. A hallmark of macrophages is their plasticity in response to their microenvironment (108). Classical activation results in immune stimulatory, pro-inflammatory cells, while alternatively activated macrophages comprise a wide spectrum of subtypes with functions in tissue repair, angiogenesis, and immune regulation. TAM activation is skewed by factors of the TME to adopt a spectrum of phenotypes that represent mixed forms of alternatively activated and pro-inflammatory macrophages (108), which has also been clearly demonstrated for TAMs in ovarian cancer ascites (12). TAMs do not possess tumoricidal activity, but are rather thought to promote immune suppression and various aspects of cancer growth and progression, including tumor cell invasion, angiogenesis, and metastasis (108) (Figure 3). Consistent with these tumor-promoting functions of TAMs, expression of the alternative activation marker CD163 in TAMs from malignancy-associated ascites showed a strong correlation with early relapse of serous ovarian carcinoma after first-line therapy (12). Among the soluble factors contributing to TAM polarization, tumor progression, and a poor clinical outcome, IL-6, IL-10, TGFβ, and AA play a prominent role (12, 40, 48–50, 109).

As shown in mouse models, normal macrophages can have two developmentally different origins. While infiltrating macrophages are derived from blood monocytes produced by the bone marrow, resident tissue macrophages, including peritoneal macrophages, are of fetal (yolk sac) origin (110–116). Although TAMs can be derived from recruited blood monocytes (117–119), tissue-resident macrophages can also be a substantial source for the generation of TAMs (112, 120–125). A recent study (109) has revealed a surprising similarity between TAMs from ovarian cancer ascites and resident peritoneal macrophages with respect to their global transcriptional profile, the expression of differentiation markers, and their activation state. For example, both resident peritoneal macrophages and TAMs are characterized by high expression of the alternative activation markers CD163 and CD206, and both TAMs and peritoneal macrophages express genes with essential functions in phagocytosis and antigen presentation at similarly high levels. The only clearly discernible difference between both cell types was an upregulation of genes linked to ECM remodeling in TAMs (109). On the other hand, blood monocytes are most likely attracted by chemokines of the tumor miroenvironment and therefore consequently contribute to the generation of TAMs. However, it remains an unresolved issue at present to which extent infiltrating monocytes and resident peritoneal macrophages represent the origins of ovarian cancer ascites-associated macrophages.

A therapeutically beneficial effect of “re-educated” TAMs has been reported for patients with pancreas adenocarcinoma and a related mouse model (126), suggesting that TAMs may also represent a potential target in ovarian cancer, as both ascites and tumor tissue typically show a high density of TAMs. TAMs from ovarian cancer ascites can indeed be shifted under experimental conditions to trigger a partial cytotoxic activity under experimental conditions (127, 128).^1^ However, the observations are partly contradictory, and activity toward autologous tumor cells has not been analyzed. It is, therefore, unclear, whether, and if so under which conditions, ovarian cancer TAMs could be (re)differentiated to macrophages with tumoricidal properties or the ability to trigger a cytotoxic response by other immune cells under clinically relevant conditions.

Interleukin-12 is a particularly interesting cytokine in the context of ovarian cancer due to its immune stimulatory antitumor effects and its inverse associations with disease progression (129–131). A hallmark of TAMs in ovarian cancer ascites is their defect to release IL-12 in response to inflammatory stimuli, which results from a transcriptional block of the *IL12B* gene encoding the p40 subunit (109, 127, 132). Another cytokine with beneficial immune stimulatory and antitumor effects in ovarian cancer patients is interferon (IFN)γ (133–136) consistent with the observation that IFNγ can prevent the skewing of monocyte differentiation by ovarian cancer ascites from immune stimulatory IL-10^low^IL-12^high^ macrophages to TAM-like IL-10^high^IL-12^low^ cells and to abrogate the suppressive effect of ovarian cancer ascites on the inducibility by TLRs ligands of IL-12 secretion (127) (see text footnote 1). As IL-12 is an essential determinant of a cytotoxic immune response, understanding the regulatory network impinging on the *IL12B* gene may be a key to the development of efficacious TAM-targeted therapies.

### Dysfunction of Other Innate Immune Cells

Besides macrophages, other myeloid cells and NK cells represent the two main subsets of innate immune cells displaying efficient yet mechanistically distinct tumor suppressive effects. Consequently, both cell types are targeted by tumor immune escape mechanisms. One prominent mechanism is the differentiation of myeloid cells to MDSCs. These cells comprise a heterogeneous population of immature myeloid cells, including immature precursors of macrophages, granulocytes, and DCs. MDSCs are capable of inhibiting both adaptive and innate immunity *via* suppression of lymphocytes and NK cell activity. Like TAMs and Tregs, MDSC significantly impact on patient survival by enhancing cancer progression and metastasis (137).

Recruitment of monocytes and their differentiation into MDSC or TAMs strongly depends on components within the TME. In ovarian cancer, the tumor-associated inflammatory mediator PGE_2_ was shown to attract MDSC into ascites. This was dependent on the induced expression of functional CXCR4 and its ligand CXCL12, facilitating migration of MDSC into the ascites (58). Direct or indirect blocking of PGE_2_ production could effectively inhibit MDSC responsiveness, providing a rationale to target PGE_2_ signaling therapeutically (58). Interestingly, tumor-infiltrating MDSC are the predominant producers of IL-10 and also depend on IL-10 to develop their immunosuppressive function *in vivo* further, confirming the fundamental role of IL-10 for the development and maintenance of a permissive TME (138). The molecular mechanisms that regulate the differentiation and function of MDSCs in cancer have been partly identified (137); however, the mechanisms that recruit MDSCs to the tumor tissue and ovarian cancer ascites remain poorly understood.

Another important key component of the innate immune system for recognizing and eliminating cancer cells is NK cells. The most important cytotoxicity receptors that mediate NK cell-dependent immunosurveillance are the CD16 receptor, the NKG2D receptor, and the natural cytotoxicity receptors, including NKp30. Defects in NK cell function such as impaired cytotoxicity/cytokine secretion, aberrant receptor and ligand expression, reduced NK cell number, or NK cell anergy are reported in malignant diseases and involved in immune escape (139). In ovarian cancer ascites, two main pathways inhibiting NKG2D (140) and NKp30 (141, 142) activity were described. First, ovarian cancer cells release the pro-inflammatory cytokine macrophage migration inhibitory factor (MIF), which stimulates tumor cell proliferation, migration, and metastasis and promotes tumor angiogenesis. MIF targets also NKG2D expression by transcriptionally downregulating NKG2D in NK cells to diminish cytotoxicity toward tumor cells (140). Second, NK cell activity in ascites is inhibited due to high levels of soluble B7-H6, one of the ligands for the NKp30 receptor. High expression of soluble B7-H6 was associated with a diminished NKp30 expression on tumor-associated NK cells and impaired NK cell activity (142). In line with this observation, lower B7-H6 expression of patients correlated with a better OS and reduced metastasis and cancer progression (141). Thus, the impaired NK cell functions in the ovarian cancer TME may contribute to immune escape, and the underlying mechanism should be further investigated.

Interleukin-18-primed NK cells release the chemokines CCL3 and CCL4 upon exposure to ovarian cancer cells to attract immature immature DCs. The NK-DC cross talk results in upregulation of CXCR3 and CCR5 ligands (CXCL9, CXCL10, and CCL5) on DCs, which in turn can recruit CD8^+^ effector T cells to the tumor environment (143). Thus, defective NK cell activation does not only affect NK-dependent killing of target cells but may also impact on the cellular interactions mandatory for the development of an antitumor immune response. A better understanding of the cross talk among innate immune cells in ovarian cancer ascites and the consequences for tumor maintenance and metastasis is one prerequisite for the development of elaborated immunotherapies.

## Impairment of the Antitumor T Cell Response

More than 10 years ago, it was notified that increased accumulation of intratumoral CD3^+^ T cells [tumor-infiltrating lymphocytes (TILs)] in ovarian carcinoma patients delayed the recurrence of the disease and was beneficial for survival (144). Other studies confirmed that TIL frequency has a prognostic value and may be important for establishing immune-based therapy regimens (145, 146). The heightened infiltration of TIL was associated with increased levels of the cytokine IFNγ, which is a prognostic factor for longer survival and has been studied in clinical trials for treating ovarian carcinoma (135, 136). Among CD3^+^ T cells, CD4^+^ T helper 1 (Th1) cells and the canonical effector CD8^+^ T cells, also termed cytotoxic T lymphocytes (CTLs), readily produce high amounts of IFNγ upon antigen re-encounter (147, 148). Therefore, it was conceivable that both CD3^+^ T cell subpopulations, Th1 cells and CTLs, contribute to the antitumor response in ovarian carcinoma by maintaining IFNγ production. However, subsequent studies revealed that higher frequencies of intraepithelial CD8^+^ T cells, expressing the α_E_ integrin subunit CD103, are associated with a better survival prognosis in ovarian cancer, suggesting that CD8^+^ T cells rather than CD4^+^ lymphocytes contribute to the antitumor effects (149–151). Instead, CD4^+^ T cells dampened the beneficial effects of CD8^+^ T cells, as the survival median of subgroups high CD4^+^ versus CD8^+^ T cell ratios was significantly shorter.

### T Regulatory Cells

**Figure 4:**
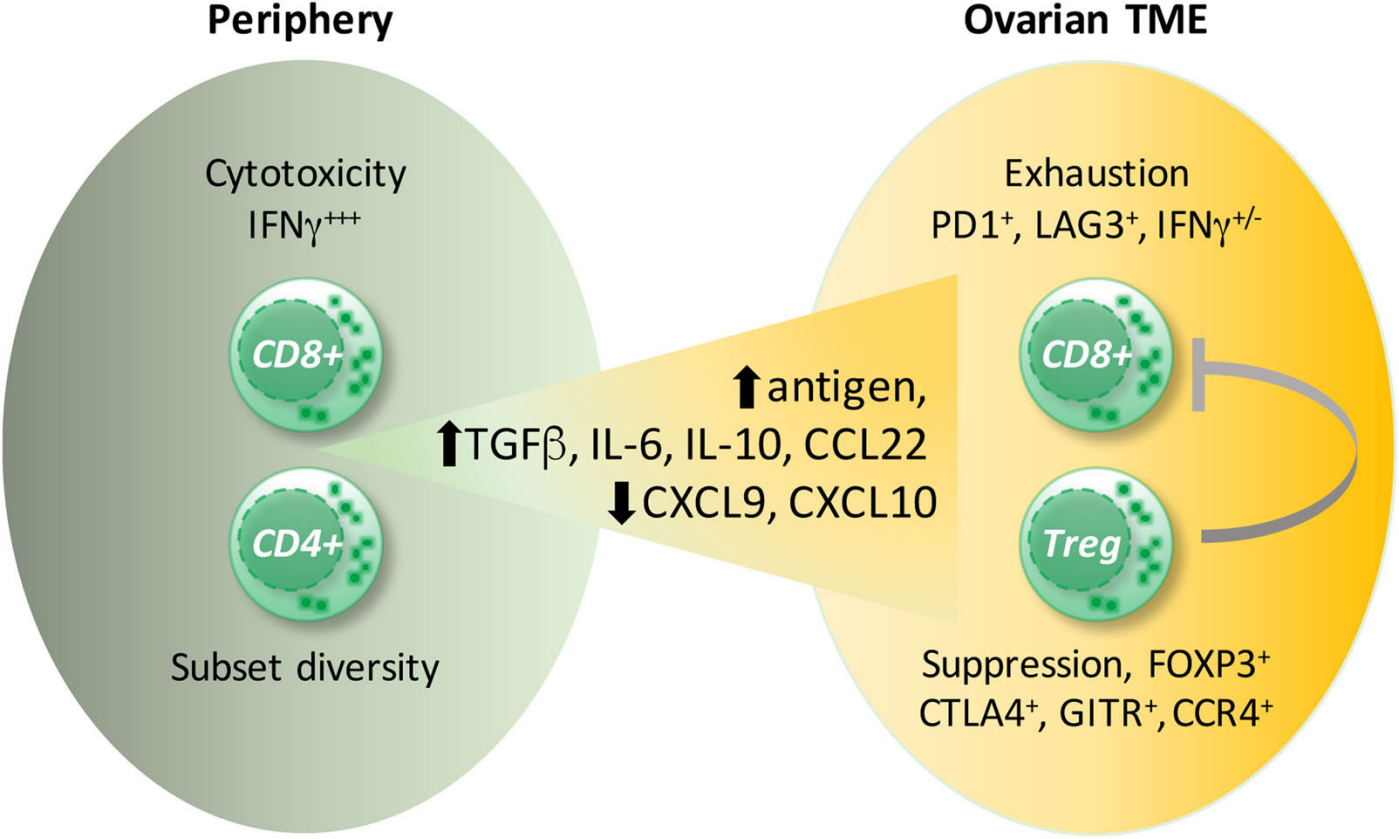
**Ovarian cancer environment and the CD8^+^ T cell versus T regulatory cells (Tregs) balance**. Signals provided by cytokines present in ovarian carcinoma ascites [interleukin (IL)-6, IL-10] as well as by dendritic cells induce exhaustion of CD8^+^ T cells. Cells coexpressing the inhibitory receptors, programmed cells death protein 1 (PD-1) and lymphocyte activation gene 3 (LAG3), display the strongest impairment in interferon (IFN)γ and TNFα production. Presumably, high antigen concentrations in the ascites cause CD8^+^ T-cell overstimulation leading to CD8^+^ T cell exhaustion. Moreover, the migration of CD8^+^ T cells into the tumor environment is diminished due to impaired expression of the T helper 1-associated chemokines CXCL9 and CXCL10. Conversely, the preferential migration of immunosuppressive FOXP3^+^CTLA4^+^, GITR^+^CCR4^+^ Tregs is supported by the upregulated chemokine CCL22.

The detrimental effect of CD4^+^ T cells was caused by an immunosuppressive subpopulation of T cells expressing the transcription factor FOXP3, termed Tregs, but not by FOXP3-negative CD4^+^ T cells (149, 152). Tregs are responsible for the maintenance of immune homeostasis by controlling autoimmunity, allergy, and inflammation, as well as responses to tumors. There is a high diversity within Tregs, dependent on their origin (thymus versus periphery), type of immune response they control, and their localization (lymphoid versus non-lymphoid tissue). Tumor-infiltrating Tregs belong to the non-lymphoid subpopulation, which differs from Tregs present in the circulation or lymphoid organs by the production of the immunesuppressive cytokines IL-10 and TGFβ as well as the expression of chemokine receptors (e.g., CCR4) and other membrane-associated immune modulators [e.g., cytotoxic T lymphocyte antigen 4 (CTLA4), GITR] (153, 154). Several reports described the presence of Tregs within ovarian cancer tumor-associated lymphocytes (152, 155, 156), and their accumulation correlated inversely with patient survival (157, 158). Tumor-associated Tregs suppressed the proliferation of, as well as IFNγ and IL-2 production by, CD8^+^ T cells specific for the tumor antigen HER2 and thereby counteracted the protective effect of tumor-specific effector T cells (158). The ovarian cancer environment caused migration of CTLA4^+^ FOXP3^+^ GITR^+^ Tregs *via* the chemokine CCL22, secreted by tumor cells and TAMs, and its receptor CCR4, expressed by infiltrating Tregs (155, 158) (Figure 4). Accordingly, the application of an anti-CCR4 antibody in an experimental model of ovarian carcinoma blocked Treg migration and enhanced antitumor response including increased IFNγ secretion by CD8^+^ T cells (159). In addition, “non-canonical” CD8^+^FOXP3^+^ cells (160) seem to be involved in ovarian cancer progression. These cells are dependent on TGFβ1 and p38 MAPK signaling (161), but their precise function remains unclear.

### Deregulation of Immune Checkpoints

Besides negative effects of Tregs, there are further factors within the ovarian cancer environment that dampen CTL-mediated antitumor responses (Figure 4). These include inhibitory pathways mediated by CTLA4, PD-1, and lymphocyte activation gene 3 (LAG3) protein (162–165). On activation *via* the T cell receptor (TCR) and costimulatory molecule CD28, which provides an essential signal for growth and survival, T cells upregulate CTLA4. CTLA4 is a negative regulator of T cell activity by competing with CD28 for the B7-1 and B7-2 ligands on antigen-presenting cells (APCs), resulting in cell cycle arrest and attenuation of effector function (166). Both, PD-1 and LAG3 are strongly upregulated by CD8^+^ T cells, and their differentiation is skewed toward a dysfunctional pathway, termed functional exhaustion (167). This state was originally described for CD8^+^ T cells arising during chronic infections upon uncontrolled antigen exposure and is characterized by gradual impairment of proliferation and production of effector cytokines IFNγ and TNFα (167). However, the exhausted CD8^+^ T cells maintain some effector function, allowing them to restrict ongoing viral replication while preventing tissue damage (168). This principle might also apply to the ovarian cancer environment in which overwhelming antigen exposure and an inflammatory milieu may cause CD8^+^ T cell exhaustion (163, 164). Nevertheless, the increased numbers of tumor-infiltrating CD8^+^ T cells correlate with a better survival prognosis (144, 149), suggesting that TILs maintain their functionality at least partially as suggested by increased levels of IFNγ in tumor tissue from patients with a favorable clinical outcome (144).

Exhaustion can be reversed by blocking signaling ligands expressed on APCs, tumor cells, or tumor-infiltrating immune cells (167, 169). Therefore, the main therapeutic focus is currently placed on the development of antibodies blocking interactions of coinhibitory receptor–ligand pairs (e.g., CTLA4:B7, PD-1:PD-L1). These antibodies are termed immune checkpoint inhibitors and have demonstrated considerable benefit in clinical trials for several cancers including ovarian carcinoma (165, 169, 170). In a mouse model of ovarian carcinoma, neutralization of CTLA4 signals caused regression of growing tumors, however, only in an early tumor stage. This was accompanied by activation of CD8^+^ T cells for IFNγ production *in vitro* (171).

Anti-CTLA4 antibodies (ipilimumab) have been administered to ovarian carcinoma patients after vaccination with irradiated, autologous tumor cells engineered to secrete GM-CSF. This treatment resulted in antitumor effects only in a minority of patients, which is in sharp contrast to melanoma (162). The histologic analysis revealed a linear relationship between tumor necrosis and an increased ratio of intratumoral CD8^+^ T cells to FOXP3^+^ Tregs (162). This finding suggests that anti-CTLA4 antibodies could influence not only effector CD8^+^ T cells but also Tregs, which constitutively express CTLA4. Indeed, anti-CTLA4 monoclonal antibody therapy is associated with depletion of intratumoral FOXP3^+^CD4^+^ Tregs and probably with elimination of immune checkpoint blockade of effector CTLs (172). However, in animal studies, the antitumor activity of anti-CTLA4 monoclonal antibodies was attributed to the depletion of Tregs rather than to the direct activation of effector CTLs (173–175). Taken together, these results suggest that the interference with the CTLA4 pathway, leading to depletion of Tregs and probably to activation of effector CD8^+^ T cells, is involved in the control of ovarian carcinoma to some extent. This points to the existence of additional mechanisms suppressing CD8^+^ T cell responses, such as the upregulation of PD-1 and LAG3 expression on TIL (163).

Besides CTLA4, PD-1- and LAG3-expressing CD8^+^ T cells specific for tumor-derived antigen NY-EASO-1 were also detectable within the ovarian carcinoma infiltrates. These molecules were upregulated on CD8^+^ T cells by IL-6 and IL-10, present in ovarian carcinoma ascites, as well as by tumor-infiltrating DCs. The strongest functional impairment in the capability to produce IFNγ and TNFα was detectable within the population of CD8^+^ T cells coexpressing both LAG3 and PD-1, an effect reversible by the simultaneous blockade of these receptors (163). The synergistic suppression of CD8^+^ T cell activity by LAG3 and PD-1 was further evaluated in a mouse model of ovarian carcinoma and revealed that physical interactions between these molecules contribute to this cooperative effect (164). The suppressive effect of ovarian cancer ascites also involves dampening of the TCR-induced activation of transcription factors NFκB and NFAT, which is crucial for T cell activation, presumably resulting from an inhibition of signal transduction upstream of PLCγ (176). Consistent with the observations that both Tregs and inhibitory molecules contribute to the immune suppression in ovarian carcinoma, high levels of miR-424(322), which targets the PD-1:PD-L1 and CTLA4:B7 pathways, displayed a synergistic antitumor effect with chemotherapy by activating CD8^+^ T cells and reducing Treg infiltration in the mouse model (177).

The blockade of PD-1 by anti-PD-1 antibody (nivolumab) in patients with platinum-resistant ovarian cancer showed encouraging results (165). In addition to suppressing the activity of infiltrating CD8^+^ T cells, the migration of these cells into the TME is prevented by epigenetic suppression of Th1-associated cytokines CXCL9 and CXCL10 (178). Thus, epigenetic regulators of Th1-chemokine production caused increased migration of CD8^+^ T cells into the tumor environment associated with tumor reduction and improved the antitumor efficacy of immune checkpoint blockade in response to anti-PD-L1 antibodies in a mouse model (178). These data indicate that regulation of both the influx and function of CD8^+^ T cells in ovarian carcinoma need to be taken into account for the development of more efficacious therapies. Taken together, the suppression of the antitumor activity of CD8^+^ T cells in the ovarian cancer environment seems to be evoked by multiple mechanisms, which include (i) the inhibition of CD8^+^ T cell migration, (ii) the induction of inhibitory molecules on CD8^+^ T cells, and (iii) modulated CD8^+^ T cell activity by increased presence of Tregs.

### Role of Glucose Metabolism

There is accumulating evidence in experimental models for an influence of the TME on the glycolytic metabolism in CD8^+^ T cells, which supports proliferation and effector function (179). The competition for nutrients between tumor cells and T cells has a crucial impact on cancer progression. Thus, decreased availability of glucose in the TME suppressed IFNγ production and antitumor function of CD8^+^ T cells along with reduced glycolysis (180). Likewise, restriction of glycolysis led to insufficient production of phosphoenolpyruvate and defects in Ca^2+^-NFAT signaling, thereby suppressed antitumor CD8^+^ T cell function (181). Moreover, elevated extracellular potassium concentrations in the tumor environment dampened CD8^+^ T cell antitumor function *via* impairment of signaling pathways linked to metabolism (182). Therefore, elucidating the influence of the ovarian cancer environment on CD8^+^ T cell metabolism might extend our understanding on immunosuppression and give new insights for therapy strategies.

## Tumor Cell Adhesion and Invasion

### Role of Mesothelial Cells and the ECM

The metastatic spread of ovarian cancer cells depends on the invasion into the mesothelium, a mostly squamous epithelium that covers all organs within the peritoneal cavity. The mesothelium is formed by a single layer of mesothelial cells with an underlying basement membrane composed of collagen, fibronectin, and laminin. An early event of metastasis involves the attachment of ovarian cancer cells present in the ascites to abdominal organs. It has been shown that cancer cells attach less firmly to mesothelial cells than to the basement membrane (183) and that metastatic sites are typically devoid of mesothelial cells (183, 184). It is therefore widely believed that mesothelial cells act as a barrier for cancer cells and represent the first line of defense against ovarian cancer and against other abdominally metastasizing cancers. It is well conceivable that ovarian cancer cells attach to ECM at the site of pre-existing lesions of the mesothelial cell layer, which might occur spontaneously at low frequency or might be fostered by the inflammatory environment of the ascites. However, there is no experimental evidence for this scenario so far.

Alternatively, ovarian cancer cells can attach directly to mesothelial cells, mainly through β1 integrin (185–187) and CD44 (186, 188, 189). This attachment to mesothelial cells is favored by a genetic program of ovarian cancer cells with upregulation of mesenchymal genes, including *TWIST1* and *ZEB1* (190), as well as downregulation of epithelial genes like *CDH1* encoding E-Cadherin (191). The downregulation of E-Cadherin induces upregulation of α5β1 integrin, which mediates selective binding to fibronectin (192), providing a mechanistic basis for the enhanced ability to attach to mesothelial cells (191).

During the metastatic process, ovarian cancer cells and mesothelial cells mutually influence their behavior in multiple ways. Upon contact with mesothelial cells, ovarian cancer cells upregulate expression of the MMP-2 to cleave fibronectin and vitronectin, with the resulting fragments of fibronectin and vitronectin providing enhanced attachment to mesothelial cells through the integrins α5β1 and αvβ3 (193). The binding of α5β1 integrin on cancer cells to fibronectin on mesothelial cells promotes the activation of myosin in the ovarian cancer cells (194). As a result, the ovarian cancer cells exert myosin-dependent mechanical forces on the mesothelial cell layer to promote the disruption of mesothelial intercellular junctions and retraction of mesothelial cells leading to gaps within the mesothelial cell layer that can be utilized by ovarian cancer cells to invade into the submesothelial matrix (184, 194). This process, by which ovarian cancer cells impact on the integrity of the mesothelial cell layer, has been referred to as “mesothelial clearance” (194). As an alternative to invading through gaps in the mesothelial monolayer by inducing mesothelial retraction, ovarian cancer cells might gain access to the submesothelial tissue by actively killing mesothelial cells. Such a mechanism involving the expression of FAS-L on cancer cells and FAS on mesothelial cells has been shown for colon cancer cells *in vitro* (195).

In disagreement with the concept that mesothelial cells shield the underlying ECM from invasion by ovarian cancer cells, prometastatic functions of mesothelial cells have also been described. Ovarian cancer cells secrete TGFβ1 to activate a TGFβ1/RAC1/SMAD-mediated signaling pathway in mesothelial cells that results in secretion of fibronectin by mesothelial cells and increased ovarian cancer cell adhesion, invasion, and proliferation (196, 197).

**Figure 5:**
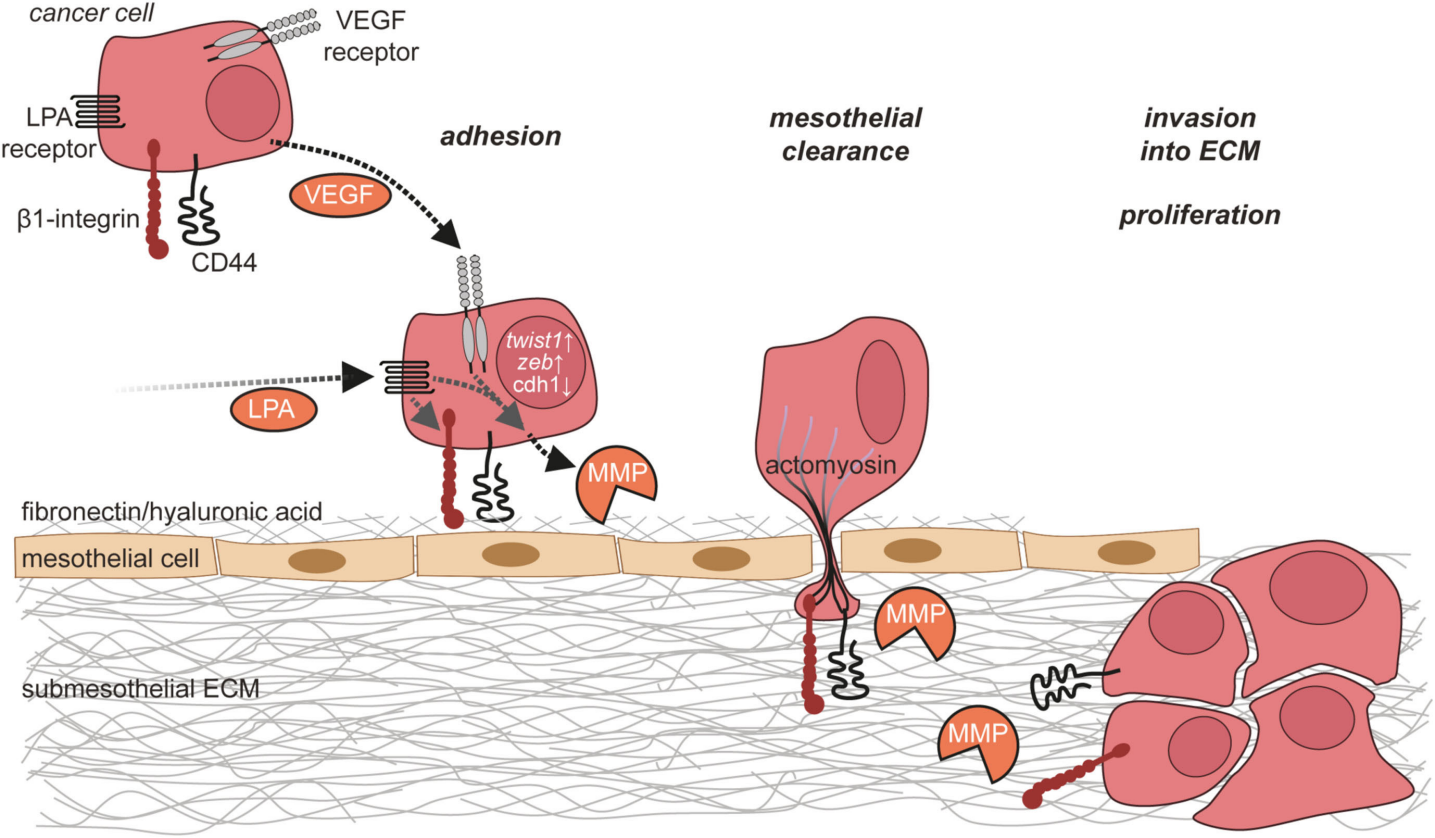
**Ovarian cancer cell adhesion and invasion**. Ovarian cancer cells in the ascites adhere to the mesothelium *via* integrins and CD44. This adhesion is further enhanced by matrix metalloproteinase (MMP)-mediated cleavage of fibronectin on mesothelial cells. Ovarian cancer cells then break mesothelial junctions to invade through the mesothelial cell layer into the submesothelial extracellular matrix (ECM), where they proliferate and form metastases.

**Figure 6:**
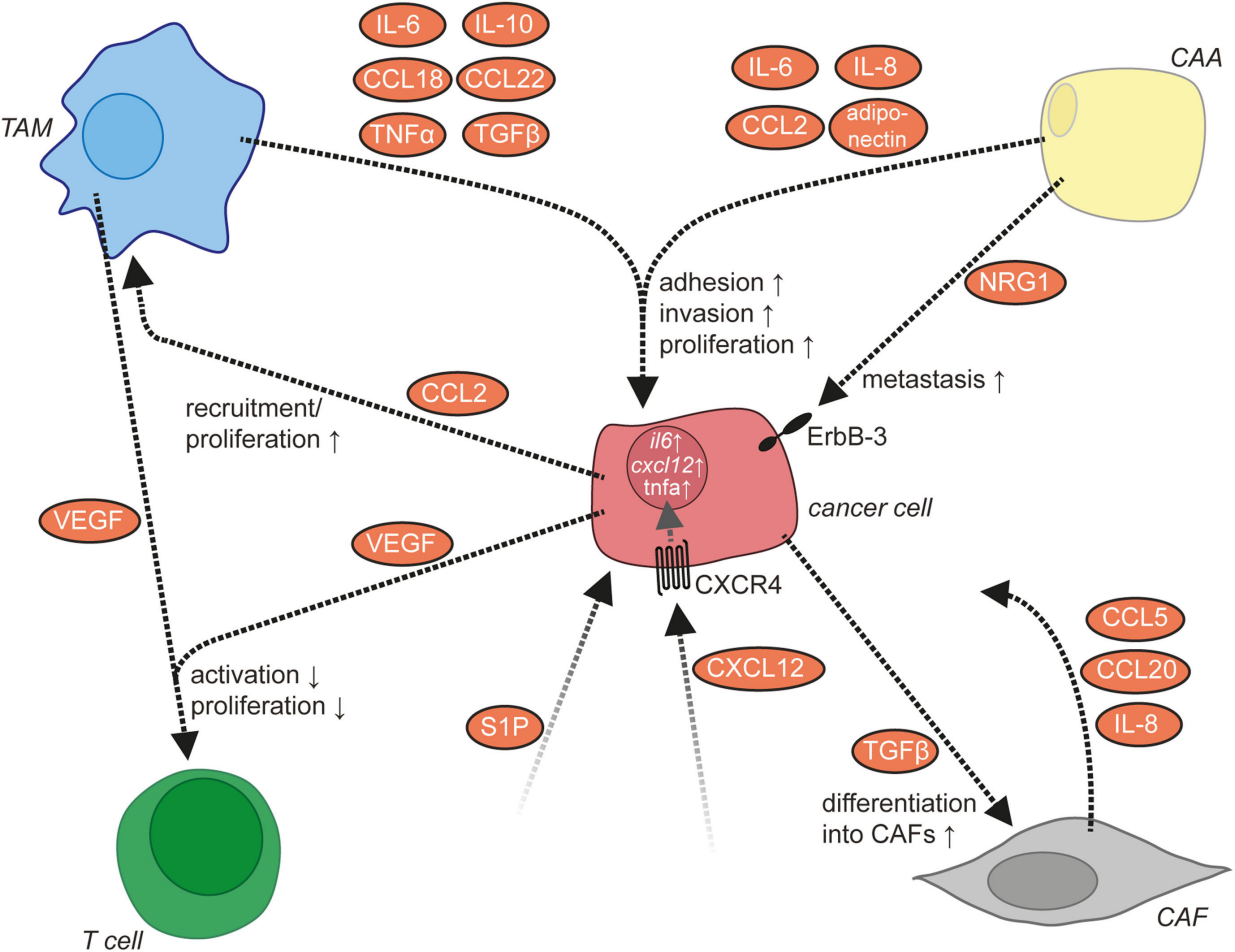
**Molecular signaling network in the ascites**. In addition to cancer cells, the ascites contains several other cell types including tumor-associated macrophages (TAMs), cancer-associated adipocytes (CAAs), cancer-associated fibroblasts (CAFs), and T lymphocytes. These cells communicate *via* multiple signaling molecules that promote the metastasis of ovarian cancer cells.

After passing through the mesothelial cell layer, ovarian cancer cells adhere to the submesothelial basement membrane, locally disrupt the basement membrane, and invade into the underlying connective tissue (184) (Figure 5). This involves the activation of MET in ovarian cancer cells, which is triggered by binding of α5β1 integrin on cancer cells to fibronectin (198). Several lines of evidence strongly suggest that TAMs contribute to the adhesion and invasion of ovarian cancer cells by promoting the remodeling of ECM (199). A comparison of the transcriptomes of ovarian cancer TAMs to that of peritoneal macrophages from non-cancer patients revealed ECM remodeling genes as the main difference between these cell populations (109). The clinical significance of this observation is supported by a strong correlation of poor survival with the expression of these genes in the PRECOG data set (109, 200). The molecular basis of the cross talk of ovarian cancer cells with cells of their microenvironment (Figure 6), in particular with immune cells such as TAMs and T cells in the ascites, and its impact on ovarian cancer cell adhesion and invasion are just beginning to be understood. In the following, we will summarize data on metastasis-promoting factors in the ascites and the molecular signaling pathways induced by them.

### Metastasis-Promoting Factors in Ascites

A large body of evidence indicates that LPA, which is frequently present in the ascites at high levels, plays a major role in ovarian cancer metastasis (see The Lipid Signaling Network of Ovarian Cancer Ascites). LPA acts *via* activation of LPA receptors on ovarian cancer cells, the expression of which is upregulated under hypoxic conditions that are frequently encountered in the TME (77). The effects of LPA on adhesion, migration, and invasion of ovarian cancer cells are mediated by multiple intracellular signaling pathways downstream of LPA receptors as described above. Mechanistically, LPA increases the expression of β1 integrin on ovarian cancer cells with enhanced adhesion to collagen as well as of MMPs and promotes the degradation of and invasion into the ECM (201). Along the same lines, LPA also induces the downregulation of tissue inhibitor of metalloproteinases expression (89). Moreover, LPA has been shown to suppress senescence of ovarian cancer cells through LPAR_2_ in the presence of wild-type p53 (202) and to induce the expression of VEGF (203).

Vascular endothelial growth factor plays a central role in neoangiogenesis, progression, and metastasis of numerous types of cancer. In ovarian cancer, VEGF is found in both the primary tumor and the ascites at high levels, and VEGF expression correlates with poor survival (204–206). Accordingly, bevacizumab, a monoclonal antibody targeting VEGF, has been approved for the treatment of advanced and relapsing ovarian cancer and prolongs progression-free survival (207). Ovarian cancer cells and TAMs constitute major sources of VEGF (52, 208) (Figure 1), but other host cells might also contribute to VEGF production (209–211). The expression of VEGF is induced by hypoxia (212, 213) and multiple other signaling molecules (214). VEGF supports ovarian cancer metastasis by acting on both ovarian cancer cells in an autocrine or paracrine manner and on other cell types including peritoneal ECs and immune cells (see below). The invasion-promoting effect of VEGF might be mediated, at least in part, by an upregulation of MMPS in ovarian cancer cells (215, 216). In addition to direct effects on cancer cells, VEGF promotes a permissive environment for ovarian cancer cell metastasis by acting on peritoneal ECs to promote angiogenesis and vascular permeability, leading to the formation of ascites (47). At sites of metastatic lesions, VEGF-induced angiogenesis is likely to support the growth of larger metastatic nodules. Moreover, VEGF might further contribute to ovarian cancer metastasis by affecting immune cell functions as there is evidence suggesting that VEGF suppresses T cell activation and proliferation (211) and that VEGF levels inversely correlate with CD3+CD56+ NK-like T cell numbers (217).

### Role of TAMs and Adipocytes in Invasion

Multiple aspects of ovarian cancer biology, including cancer cell adhesion and invasion, are under the control of a highly complex communication network between different cell types within the ovarian cancer TME. For some cytokines, ovarian cancer cells or immune cells have been identified as sites of production (Figure 1), while for other cytokines, the cell types releasing them remain unknown. TAMs represent a central player in the ovarian cancer cytokine network to support ovarian cancer cell adhesion and invasion (8). They secrete multiple metastasis-promoting cytokines including IL-6, IL-10, CCL18, CCL22, TNFα, and TGFβ (218). The number of TAMs is positively regulated by CCL2/MCP-1, which is released from ovarian cancer cells into the ascites (219). IL-6 released by TAMs promotes the adhesion, invasion, and proliferation of ovarian cancer cells (51, 220), which might be mediated in part by an increase of MMP expression by ovarian cancer cells (220). TNFα produced by TAMs also promotes invasion of ovarian cancer cells, the exact mechanism of which awaits further clarification (221, 222). Through the release of CCL22, TAMs attract Tregs to ovarian cancer cell clusters, which in turn suppress cytotoxic T cells (158).

CXCL12/SDF-1 released by an unknown cellular source into the ascites acts on cancer cells *via* CXCR4 (223). The expression level of CXCR4 is elevated by binding of ovarian cancer cells to ECM *via* β1 integrin (223). The activation of CXCR4 results in the upregulation of the prometastatic cytokines IL-6, CXCL12, and TNFα in ovarian cancer cells (8) and increases metastasis (224). IL-6 secretion by ovarian cancer cells is also increased by platinum-based chemotherapy and promotes the polarization of TAMs toward a protumorigenic and chemoresistant phenotype (225). Another mediator present in the ascites is the lipid sphingosine-1-phosphate (SIP), which stimulates invasion of ovarian cancer cells, probably through several different signaling pathways downstream of SIP receptors (226–229).

In addition to ovarian cancer and immune cells, adipocytes of the omentum are actively involved in cytokine signaling. They secrete IL-6, IL-8/CXCL8, CCL2, and adiponectin, which act on cancer cells *via* their respective receptors to support ovarian cancer cell metastasis (230). Adipocytes thereby establish a specific microenvironment with strong growth-promoting effects on disseminated ovarian cancer cells. Examples in this context are (i) the promotion of tumor cell homing and invasion into the omentum by adipocyte-derived IL-8 (230) in cooperation with CAFs, (ii) the direct transfer of fatty acids from adipocytes to adjacent tumor cells *via* fatty acid-binding protein 4 for energy production (230), and (iii) the activation of the ERBB3 receptor on metastasizing cancer cells by the mitogenic ligand neuregulin 1 (5).

## Stat and NFκB Signaling Networks: Opposing Cell Type-Specific Functions

### Similarity of the TME with the Resolution Phase of Wound Healing

Besides tumor cells, large populations of Tregs, TAMs, and possibly other host cells provide high levels of immunosuppressive mediators, thereby strongly limiting the fraction of cells competent to produce the crucial pro-inflammatory cytokines, such as IFNγ and IL-12. The influx of potentially cytotoxic T cells, monocytes, and NK cells cannot counteract this efficient tumor protection scheme: the dominant TME at advanced stages of the disease converts their phenotypes toward tumor promotion and immune suppression despite the presence of pro-inflammatory molecules, including cytokines, lipids, and nitric oxide as well as cellular debris and discharged intracellular molecules acting as ligands for TLRs (231, 232). This cocktail exerts strong suppressive functions on all types of immune cells, but at the same time promotes the protumorigenic properties of cancer cells, enabled by cell type-specific signal transduction mechanisms. It has been known for a long time that tumors resemble wounds that do not heal (233, 234). The final stage of wound healing, termed resolution phase, provides effective termination of inflammation. Therefore, it is tantalizing to speculate that the TME of advanced tumors lock immune cell types in a state resembling a never-ending resolution phase, while deregulated pro-inflammatory, protumorigenic signaling persists in the tumor cells. Consistent with this idea is the observation that ovarian cancer TAMs differ from normal peritoneal macrophages mainly by the upregulation of ECM remodeling genes (109), which is also the hallmark of resolution phase (235). Moreover, type I, III, and V collagens are major components of the TME, similar to a healing wound (233). These collagens are also among the matrix proteins upregulated in ovarian cancer TAMs, and their expression shows a striking association with poor survival (109).

### Cell Type-Specific Effects by Mediators of the TME *via* Differential Signal Integration

**Figure 7:**
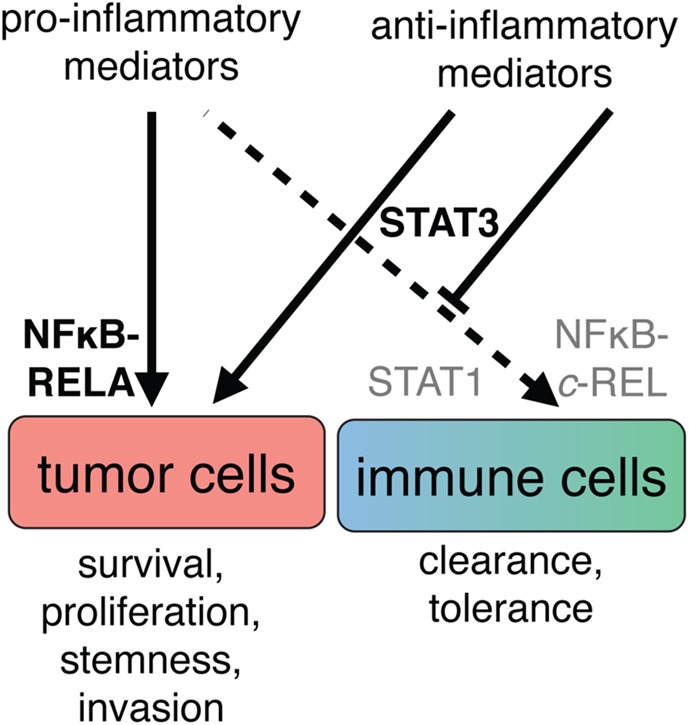
**A simplified view of the cell type-specific impact of cytokine signaling in the ovarian cancer tumor microenvironment**. The simultaneous presence of pro- and anti-inflammatory mediators suggests that biological outcome depends on both prevalence and interpretation of signals. Left: tumor cells respond with persistent activation of the transcription factors RELA (p65) and STAT3, leading to promotion of growth, progression, and poor clinical outcome. Right: cells of the immune system do not receive appropriate combinations of stimuli for pro-inflammatory activation and therefore do not activate REL and STAT1. Integration of the signaling molecules present in ascites leads to an anti-inflammatory state, at least in part through activation of STAT3.

A vast number of studies attribute the activation states of both innate and adaptive immune cells to differential activation of JAK/STAT and NFκB signaling. The JAK/STAT network is primarily regulated by cytokines that have either pro- or anti-inflammatory properties (236) and convey intercellular communication. NFκB proteins are effectors of receptors that detect either host-elicited or pathogen-derived signals and trigger mostly pro-inflammatory responses (237). In cancer cells, the very same networks exert mostly protumorigenic functions due to differential and constitutive activation of effector molecules and regulation of target genes. Thus, in cancer cells, STAT and NFκB have been implicated in the stimulation of proliferation, suppression of apoptosis, angiogenesis, promotion of invasion, and other protumorigenic processes (238). Due to the simultaneous presence of a plethora of both pro- and anti-inflammatory mediators, the ovarian cancer TME represents an excellent model to study how differential signal integration mediates cell type-specific biological effects (12, 14–16, 39, 52, 239). The following paragraphs describe the molecular mechanisms that, in a simplified view, enable unhindered tumor cell growth, driven by constitutive NFκB and STAT3 signaling, and simultaneously preclude immune cell activation by preventing pro-inflammatory NFκB *via* STAT3-driven mechanisms (Figure 7).

### The IL-12 Paradigm

**Figure 8:**
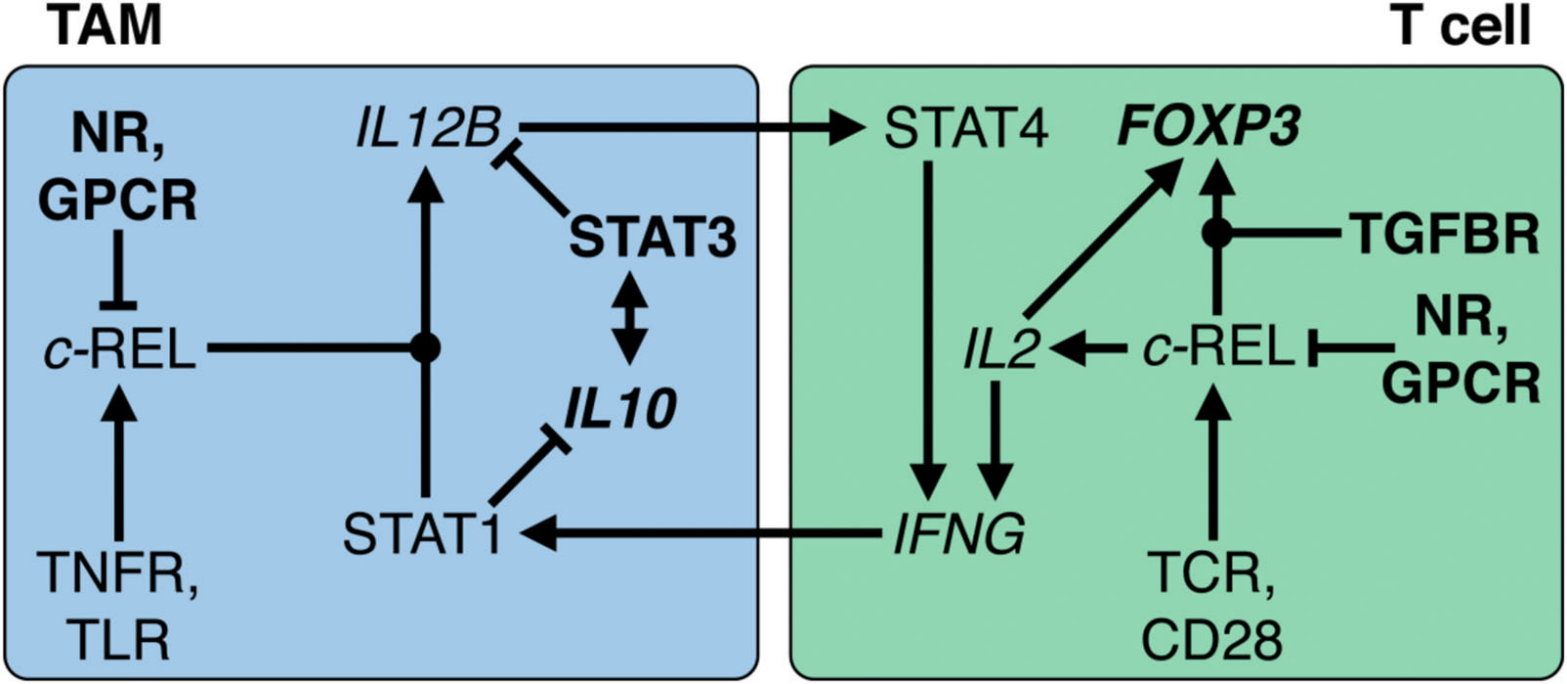
**Transcriptional circuitry governing the immunesuppressive state of macrophages and T cells in the ovarian cancer tumor microenvironment (TME)**. In tumor-associated macrophages (TAMs), interleukin (IL)-10 activates STAT3, which in turn stimulates *IL10* transcription. Both cooperate to repress *IL12B* transcription and REL nuclear translocation by multiple mechanisms, reinforced by GPCR ligands and nuclear receptor (NR) agonists such as glucocorticoids and polyunsaturated fatty acids. In T cells, REL plays an ambivalent role by fostering both *IL2* and *FOXP3* transcription. The latter depends on the presence of TGFβ and results in conversion into a T regulatory cell phenotype. In the ovarian TME or ascites, TNFR, toll-like receptor (TLR), T cell receptor (TCR), and CD28-activating stimuli are overruled; without long-term pro-inflammatory stimulation, upregulation of *IFNG* transcription is prevented. As a consequence, pro-inflammatory signals fail to activate STAT1, induction of *IL12B*, and subsequent sustained production of interferonγ. Dot-shaped nodes denote a requirement for both signals (logical AND).

Regulation of IL-12 expression is a paradigm for the cross talk of NFκB and STAT signaling (Figure 8). In humans, macrophages exert their tumoricidal activity largely by activating NK cells *via* the secretion of IL-12 and IL-18 (240). Likewise, macrophages and other APCs use IL-12 to promote the differentiation of naïve T cells to cytotoxic Th1 cells. Occupation of the IL-12 receptor on T and NK cells induces STAT4 phosphorylation, leading to expression of IFNγ (241), a crucial activator of macrophages and class II major histocompatibility complex gene expression and thus an essential player in tumor surveillance (242, 243). IL-12 exerts its pivotal role in antitumor immune surveillance as a heterodimer of p35 and p40, encoded by the *IL12A* and *IL12B* genes, respectively. Expression of *IL12B* is under stringent transcriptional control and represents the rate-limiting step for IL-12 production (244).

Transcription of *IL12B* in macrophages requires the cooperative action of two transcription factors activated by different signaling pathways, i.e., the NFκB subunit REL and STAT1. While NFκB activation is triggered by pro-inflammatory cytokines and TLR ligands, STAT1-containing dimers are induced by interferons (245). STAT1 acts to orchestrate remodeling of promoter-proximal nucleosomes as a prerequisite for activation of *Il12b* transcription by NFκB proteins (246). Thus, the sequential action of STAT1 and REL in macrophages is crucial for the establishment of a cytotoxic immune response that depends on IL-12 production by macrophages. This provides for a highly efficient control mechanism that physiologically safeguards against detrimental inflammatory reactions in the absence of either activated STAT1 or REL. Tumors exploit this stringent regulatory network through signaling mediators that prevent the simultaneous activation of both pathways. IL-12 production is efficiently suppressed by numerous molecules found in ovarian cancer ascites, including IL-10, PS, fatty acid derivatives, and G-protein receptor ligands (247–259). The underlying mechanisms include both global and gene-specific suppression of the NFκB response in macrophages.

### Functions of REL in T Cells

Apart from its role in APCs, REL is required for the expression of *IL2* in T and NK cells and thereby necessary for their proliferation, survival, and activation (Figure 8). This includes the production of IFNγ, thus establishing a positive feedback loop between both cell types. Importantly, transcription of both *IL12B* and *IL2* is fully dependent on REL; its function cannot be substituted by other NFκB proteins (260–262). However, dependent on the microenvironment, activation of REL in T cells can also dampen the immune response, e.g., in the presence of TGFβ, as is the case in ovarian cancer ascites. Thus, a number of mouse studies showed that the TGFβ-activated SMAD3 transcription factor cooperates with REL to induce expression of *FOXP3* and that IL-2 contributes to *FOXP3* induction by activating STAT5 (263, 264). As a consequence, FOXP3 drives the differentiation of naïve CD4-positive T cells to Treg, thereby preventing IFNγ production. These mechanisms are physiologically important to enable states of immune privilege or tolerance, which ensure that initiation of inflammation is efficiently prevented in reproductive organs, brain, and eyes. This regulatory network orchestrated by immunosuppressive macrophages and Tregs (265) is the blueprint for the immunesuppressive mechanisms invoked in the ovarian cancer TME.

### Persistent STAT and NFκB Activation in Tumor Cells

In contrast to immune cells, NFκB is constitutively active in >50% of ovarian carcinomas and is correlated with poor survival (266–268). In fact, inflammatory signaling involving NFκB and STATs has been implicated in the genesis of many tumor entities. Activation of NFκB in cancer cells is most frequently caused by mediators of TME, since mutations of NFκB factors are confined to lymphoid malignancies (269). In ovarian cancer ascites, these mediators include IL-6, TNFα, as well as TLR and EGF receptor ligands (8, 12, 15, 52, 270). Proteins encoded by NFκB target genes include anti-apoptotic, growth-promoting, proinvasive, proangiogenic and immunesuppressive proteins, exemplified by BCLX-L, IL-6, IL-8, VEGF, and PD-1L, which explains its multifaceted role in ovarian cancer progression and therapy resistance (268, 271). Platinum-based chemotherapy also induces NFκB in ovarian carcinoma (272). Strikingly, RELA was found among the upregulated proteins in carboplatin-resistant tumors, as well as STAT5B (253). STAT5 and STAT3 are frequently hyperactive in human cancers, driving apoptotic blockade, invasion, stemness, and pro-inflammatory signaling, at least in part through persistent NFκB activation (273–275).

### Drugging NFκB and STAT Networks

NFκB signaling impacts both protumorigenic and antitumorigenic mechanisms in different cell types; while enabling unhindered growth of tumor cells, it is also crucial for innate and adaptive immune responses as exemplified by its requirement for induction of *IL12B*. Accordingly, cautionary advice has been worded regarding broad pharmacological intervention (252, 256). Non-selective targeting, for example, inhibition of proteasome activity or bromodomain-mediated acetyl-lysine binding, has been tested in clinical studies, but it remains unclear whether these compounds have long-lasting benefits. Drugs that target factor(s) with protumorigenic roles in both tumor and stromal cells may be more promising targets. STAT3 was proposed as a common factor mediating negative effects (274, 275). However, caveats with the development of STAT3 antagonists are its high homology with STAT1 and its roles in barrier function and host defense, and considerable efforts dedicated to the development of specific and bioavailable STAT antagonists have met with little success (258). To circumvent the multifaceted roles of signaling networks, cell type-specific signaling will have to be identified and targeted to avoid the pitfalls of previous drug development strategies. For example, counteracting IL-10 (and other, currently unknown) TME-mediated immunosuppressive signaling events and thereby allowing for STAT1 activation, c-REL translocation, and IL-12 production might be a promising approach.

## Author Contributions

All authors contributed significantly to this review article with a focus on specific topics. TW: cancer cell adhesion and invasion; ES: EVs and NK cells; MH: T cells; TA: NFκB and STAT signaling pathways; UW: clinical aspects; SR: intercellular signaling; RM: all other topics, conceptual design, and coordination. All authors read and approved the manuscript.

## Conflict of Interest Statement

The authors declare that the research was conducted in the absence of any commercial or financial relationships that could be construed as a potential conflict of interest.

## Footnotes

^1^Adhikary T, Wortmann A, Finkernagel F, Lieber S, Nist A, Stiewe T, et al. Interferon signaling in ascites-associated macrophages is associated with a favorable clinical outcome in a subgroup of ovarian carcinoma patients. *BMC Genomics* (2016) (submitted).

## Abbreviations

APC, antigen-presenting cell; CAA, cancer-associated adipocyte; CAF, cancer-associated fibroblast; DC, dendritic cell; EC, endothelial cell; EV, extracellular microvesicle; IFN, interferon; IL, interleukin; HGSOC, high-grade serous ovarian carcinoma; MDSC, myeloid-derived suppressor cell; NK cell, natural killer cell; SIP, sphingosine-1-phosphate; STAT, signal transducer and activator of transcription; STIC, serous tubal intraepithelial carcinoma; TAM, tumor-associated macrophage; TGFβ, transforming growth factor β; TIL, tumor-infiltrating lymphocyte; TLR, Toll-like receptor; TME, tumor microenvironment; Treg, T regulatory cell; VEGF, vascular endothelial growth factor.
